# Target Essentiality and Centrality Characterize Drug Side Effects

**DOI:** 10.1371/journal.pcbi.1003119

**Published:** 2013-07-11

**Authors:** Xiujuan Wang, Bram Thijssen, Haiyuan Yu

**Affiliations:** 1 Department of Biological Statistics and Computational Biology, Cornell University, Ithaca, New York, United States of America; 2 Weill Institute for Cell and Molecular Biology, Cornell University, Ithaca, New York, United States of America; 3 Department of Bioinformatics, Maastricht University, Maastricht, The Netherlands; National University of Singapore, Singapore

## Abstract

To investigate factors contributing to drug side effects, we systematically examine relationships between 4,199 side effects associated with 996 drugs and their 647 human protein targets. We find that it is the number of essential targets, not the number of total targets, that determines the side effects of corresponding drugs. Furthermore, within the context of a three-dimensional interaction network with atomic-resolution interaction interfaces, we find that drugs causing more side effects are also characterized by high degree and betweenness of their targets and highly shared interaction interfaces on these targets. Our findings suggest that both essentiality and centrality of a drug target are key factors contributing to side effects and should be taken into consideration in rational drug design.

## Author Summary

The ultimate goal of medical research is to develop effective treatments for disease with minimal side effects. Currently, about 20% of drug candidates failed at clinical trial phases II and III due to safety issues. Therefore, understanding the determining factors of drug side effects is of paramount importance to human health and the pharmaceutical industry. Here, we present the first systematic study to uncover key factors leading to drug side effects within the framework of the human protein interactome network. Our results show that it is the number of essential targets, not the number of total targets, of a drug that determines the occurrence of its side effects. Furthermore, we find that the centrality, both degree and betweenness, of the drug targets is also an important determining factor of drug side effects. Our findings will shed light on new factors to be incorporated into the drug development pipeline.

## Introduction

Regardless of their effectiveness, most drugs come with side effects of different types that affect patients' life quality and may even bring up additional health problems. It is estimated that around two million patients suffer from serious drug side effects each year and that the fourth leading cause of death in the United States is severe side effects of medication [1], [2]. Of the total number of drug candidates failed during clinical trial phases II and III, 20% of these failures are because of safety issues [3]. Hence, evaluating potential side effects of drugs is important in rational drug design and development, as well as successful marketing. Binding of drugs to their on- and off-targets modifies the functions of these targets and therefore is believed to account for their efficacies as well as side effects [4]. Traditionally, properties of a drug such as binding fingerprint and chemical structure are evaluated to anticipate side effects [5], [6]. Moreover, *in vitro* assays or phenotypic tests in model organisms may not be able to capture the same spectrum of side effects in human [7], [8].

Recently, an increasingly accepted view is that integrating biological networks would provide unique insights into understanding disease mechanisms and identifying novel drug targets [9], . Network-based methods have been explored and successfully applied in finding disease-associated genes and inferring underlying molecular mechanisms [11], [12]. Similarly, phenotypic responses to drugs can be better rationalized by considering their overall effects in the context of molecular networks. Previous studies have shown that drugs with shared targets or those that are close in the interactome network often share similar side effects [13], [14]. Also, similar side effect profiles have been used to predict drug-target interactions for potential drug repositioning [13]. Hase *et al.* examined network degree distribution of different categories of genes and suggested that connectivity is potentially important in inferring drug side effects [15]. However, no actual adverse effect data were used in their study. The relationships between drug target properties, especially in the context of biological networks, and its potential toxicity to human remains unexplored. Here, we systematically investigate major contributing factors of drug side effects, taking into consideration their direct targets and the local network structures of these targets.

## Results/Discussion

**Figure 1:**
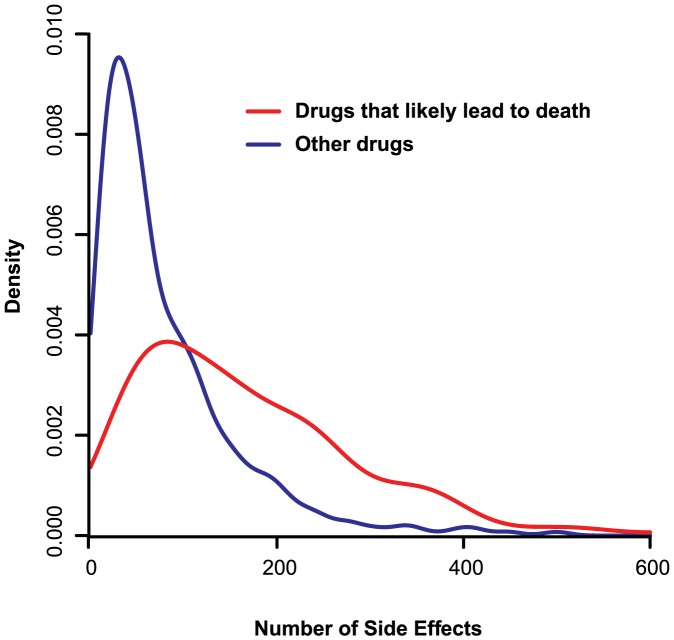
Drugs causing more side effects tend to be associated with more severe outcomes including death. Drugs were classified into two groups: 1) drugs that have a reported side effect described as “death” in SIDER 2 (red) and 2) drugs that do not have a reported fatal side effect (blue). The number of side effects for drugs more likely to lead to death has a right-shifted distribution.

**Figure 2:**
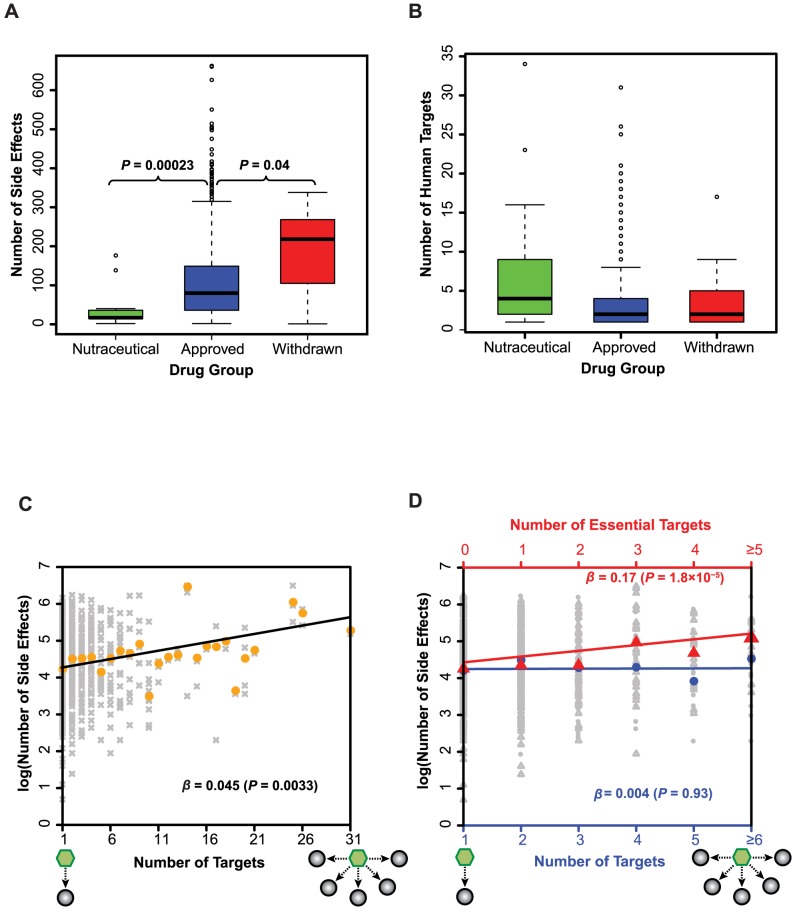
The number of side effects is positively correlated with the number of essential targets. (**A**) The number of side effects and (**B**) the number of human targets are displayed for different groups of drugs. The Wilcoxon rank-sum tests were used to assess the differences in distributions of side effects and human targets among different drug groups. The number of drug side effects is positively correlated with (**C**) the number of total targets and (**D**) the number of essential targets (triangles). However, the analysis on drugs with no essential targets shows no correlation between drug side effects and targets (circles). The results in panels (C) and (D) are obtained from generalized linear regressions based on negative binomial distribution for side effects. In panels (C) and (D), gray symbols are raw data while the colored ones correspond to median counts of side effects. Schematics under the x-axes illustrate a drug (hexagon) binding to its target protein(s) (filled circles).

We obtained a list of 996 drugs and the associated 4,199 side effects from SIDER 2 [16] and analyzed 645 FDA-approved drugs that have at least one known human protein target based on the DrugBank database [17]. Evaluation of severity of adverse effects varies among individuals and is often affected by an individual's underlying health conditions. In general, drugs that cause more side effects tend to have higher likelihood leading to severe outcomes, including death (Figure 1). Although tremendous efforts have been made on studying drug side effects in the pharmaceutical industry, the number of side effects for FDA-approved drugs significantly increases for those that were approved recently (Figure S1), indicating the necessity in further studying the contributing factors underlying drug adverse effects. By grouping drugs into the categories of “nutraceutical”, “approved”, and “withdrawn” drugs, we find that, unsurprisingly, the nutraceutical drugs have the least number of side effects (*P*-value = 0.00023, when compared to the approved therapeutical drugs; Figure 2A), while the withdrawn drugs cause significantly more side effects compared to the approved ones (*P*-value = 0.04; Figure 2A). However, there is no significant difference between the average numbers of targets of the three drug groups (Figure 2B). This indicates that the occurrence of side effects may not simply be explained by the number of targets a drug binds to. To investigate this further, we performed a generalized linear regression with negative binomial distribution for side effects over the number of targets. At first, we observed that the number of side effects significantly correlates with the number of targets (*β* = 0.045; *P*-value = 0.0033; Figure 2C). However, further dissection of properties of drug targets reveals that the positive correlation is due to the presence of essential targets, those drug targets encoded by essential genes. We find that the positive correlation between the number of side effects and that of essential targets is much more significant (*β* = 0.17; *P*-value = 1.8×10^−5^; Figure 2D). On the contrary, by analyzing drugs with no known essential targets, we find that the positive correlation between the number of side effects and targets no longer holds (*β* = 0.004; *P*-value = 0.93; Figure 2D; see Figure S2 for the illustration separating the effects of essential and non-essential targets). This discovery suggests that it is the number of essential targets, rather than the number of total targets, that governs the occurrence of drug side effects.

**Figure 3:**
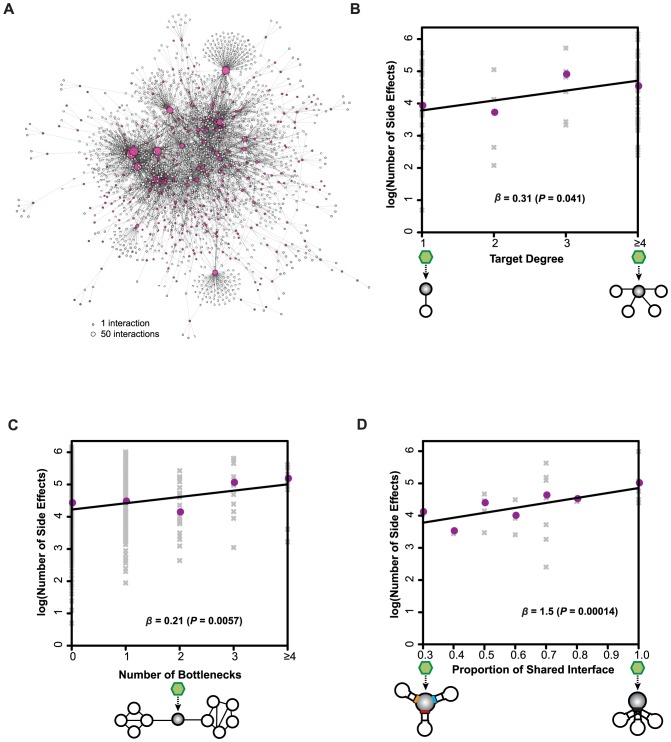
The number of side effects is positively correlated with target centrality in the protein-protein interaction network. (**A**) Network representation of the human protein-protein interactome for drug targets. Nodes represent proteins and edges correspond to interactions. Colored nodes in the panel (A) indicate the known drug targets. The number of drug side effects is positively correlated with (**B**) the degree of a target, (**C**) the number of bottleneck targets, and (**D**) the proportion of shared interaction interface on a target. All the results are obtained from generalized linear regressions based on negative binomial distribution for side effects. Gray symbols in the panels (B)–(D) are raw data while the colored ones correspond to median counts of side effects. Schematics under the x-axes illustrate a drug (hexagon) binding to its target protein(s) (filled circles): In (B), open circles represent interaction partners of the drug targets. In (C), the filled circle is a bottleneck target and open circles represent non-bottleneck proteins in the network. In (D), different interfaces of a multi-interface drug target are highlighted in colors; the interface of a single-interface drug target is highlighted in black.

Moreover, the human interactome network has been demonstrated to be highly valuable in understanding pathogenic mechanisms of many disease genes [9], since most proteins interact with other proteins to carry out their functions [18]. Therefore, it is also important to assess drug side effects by considering network properties of their targets within the human protein interactome. Here, we examined whether the degree (number of proteins that directly interact with the targets) and betweenness (number of shortest paths going through the targets) [19] of drug targets in the network contribute to side effects. These are two of the most important network parameters, measuring the centrality of the target proteins within the network. We constructed a high-quality human protein-protein interactome network that consists of 30,713 interactions between 8,357 proteins and then mapped all the drug targets onto the interactome (Materials and Methods; the sub-network containing the drug targets is shown in Figure 3A). This high-quality human protein-protein interactome network can provide insights into potential toxicity of drugs based on the network properties of their targets.

To systematically investigate the relationship between a drug's side effects and its target degree within the interactome network, we focused on drugs with only one non-essential target to separate potential confounding effects of the number of total and essential targets. The results show that the number of side effects correlates significantly with the degree of drug targets (*β* = 0.31; *P*-value = 0.041; Figure 3B). Furthermore, we analyzed the occurrence of side effects with respect to the number of targets that are bottlenecks [19] (network nodes with betweenness among top 20%) and found significant positive correlation between them (*β* = 0.21; *P*-value = 0.0057; Figure 3C). This positive correlation is consistent when we set the betweenness cutoff at top 5%, 10%, and 40% for identifying bottleneck proteins (Figure S3). This observation indicates that the centrality of drug targets in biological networks also plays a key role in producing various side effects. We further partitioned the drugs into cancer and non-cancer drugs and repeated the calculations for essentiality and centrality that we presented above. We found the same conclusions for both cancer (Figure S4) and non-cancer drugs (Figure S5).

Our recent study has shown that reconstructing the human protein interactome into a three-dimensional (3D) structurally resolved network can provide insights into molecular mechanisms of disease genes and their mutations [12]. To understand distinct perturbations of the interactome network by various drugs, we then examined the properties of their targets within the framework of our 3D-interaction network. The structural details in this 3D-interaction network allow us to distinguish the effects of drug targets with distinct binding interfaces (i.e., multi-interface targets, which bind their different interaction partners at different interfaces) and those with a common interface (i.e., single-interface targets, which bind their different partners at the same interfaces) [20]. We hypothesize that more adverse effects are expected for a single-interface target due to a higher likelihood of altering all of its interactions by a drug disrupting its only interaction interface. By analyzing side effects of a drug with the proportion of shared interaction interfaces of each drug target with its interaction partners, we observe that the number of side effects increases significantly with the proportion of shared interaction interfaces on a target (*β* = 1.5; *P*-value = 0.00014; Figure 3D). This observation confirms our hypothesis that single-interface targets are likely to cause more side effects than multi-interface ones. We show that this finding is not due to potential biases contributed by hubs or bottlenecks since these nodes tend to have smaller proportions of shared interaction interfaces (Figure S6).

**Figure 4:**
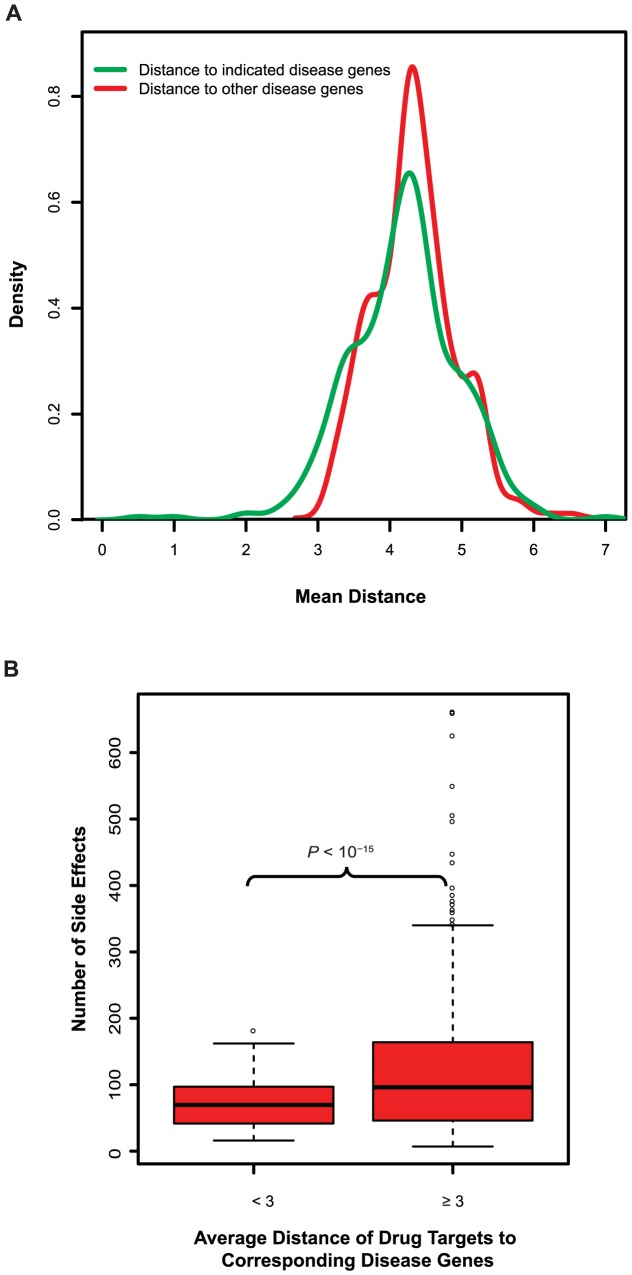
Distribution of side effects for drugs categorized by distance between drug targets and disease genes. (**A**) The distribution of distance between drug targets and their indicated disease genes highly overlaps with their distance to other disease genes. (**B**) Distribution of side effects for drugs categorized by average distances between their targets and corresponding disease genes.

We further identified genes associated with human genetic disease and mapped them onto our human protein interactome network [12]. We calculated the average shortest distances between drug targets and disease-associated genes to represent potential molecular steps needed for a drug to affect the corresponding disease module/pathway. We find that although there is an enrichment of shorter distance between drug targets and their “indicated disease” genes, the distribution largely overlaps with that of distance between targets and unrelated disease genes (Figure 4A). Furthermore, the drugs that fail to specifically interfere with the disease-associated module/pathway result in many more side effects (Figure 4B). This result further demonstrates the importance of incorporating network properties of drug targets and corresponding disease genes in rational drug design and development.

In summary, for the first time, we show that the number of essential targets, not the number of total targets, is a determinant of drug side effects. Furthermore, high incidence of drug side effects is also characterized by high degree and betweenness of their targets in the interactome network, as well as highly shared interaction interfaces on these targets. Our findings reveal that both essentiality and centrality of a drug target are important factors to be considered in the drug development pipeline in order to improve the efficiency of this lengthy and costly process. Incorporation of these factors will be useful in the selection of drug candidates at the early stages of the drug development pipeline. When choosing from several drug candidates with similar chemical properties, the one binding to proteins that are not essential and not central in the network would have a higher chance of passing clinical trials later. Moreover, in the efforts of computationally predicting drug side effects [21], the inclusion of target essentiality and centrality as additional features would also improve the prediction performance. Furthermore, our results can serve as guidance for minimizing side effects in clinical applications, especially when prescribing multi-drug cocktails, which have been proven to be much more effective than single drug approaches [22]. With the increasing coverage of the protein-protein interaction network in human and the accessibility of interactions of high confidence levels [23], more interesting analyses can be performed to further dissect the properties of drug targets and the associated side effects. This study of adverse effects of drugs within the framework of the protein-protein interactome network demonstrates that network-based pharmacology is of great importance in the field of drug development and application.

## Materials and Methods

### Compiling a comprehensive list of drug side effects, human targets, and target essentiality

We downloaded 4,199 side effects associated with 996 drugs from the SIDER database release 2 [16]. For the drugs in SIDER 2, we mapped them based on the generic drug names or PubChem IDs [24] to the DrugBank database [17] downloaded on November 6, 2011, and extracted all of their direct binding human protein targets (647 in total) with available uniprot IDs. We did not differentiate on- and off-targets in all of our analyses with the rationality that they could all potentially produce side effects when bound by the corresponding drugs. Furthermore, we downloaded the database containing the approval dates for each drug from the Drugs@FDA database (http://www.accessdata.fda.gov/scripts/cder/drugsatfda/) and the Orange Book (http://www.accessdata.fda.gov/scripts/cder/ob/eclink.cfm). The earliest approval date was used when a drug had a history of multiple approval events. We then cross-checked the list with the ones reported by Rask-Andersen *et al.* [25] and removed the drugs with conflicting dates. A list of essential genes was obtained by taking the union of the human orthologs of mouse genes that result in embryonic or postnatal lethality when disrupted [26] and the genes reported as essential from a large-scale RNAi screen in human mammary cells [27]. A drug target that belongs to the essential gene list is abbreviated as an “essential target”.

### Generalized linear regression analysis

To find key factors contributing to the incidence of side effects, we performed a series of generalized linear regressions based on negative binomial distribution for side effects with the following probability density function:
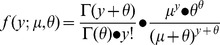
with mean *μ* and shape parameter *θ*. The expected value and variance for the number of side effects are:
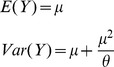



This model is used because we observed over-dispersion with Poisson distribution, which is normally modeled for count data. The generalized linear regressions were built using the log-link function:
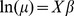
where *X* is the independent variable (such as the number of targets), *β* is the unknown parameter, and 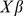 is the linear predictor. To minimize the effects of extreme observations, we used median numbers of side effects as response variables for regression analysis. For each regression, we obtained a *P*-value for the effect of a tested factor based on the hypothesis testing: H_0_: *β* = 0 (there is no effect of the tested factor) vs. H_A_: *β*≠0 (the incidence of side effects is contributed by the factor). Due to the lack of data points, a few observations at the margin were binned together. We first fitted regression for the number of side effects over that of total targets and that of essential targets. To distinguish the effect of total targets and essential targets on the incidence of side effects, we repeated the regression analysis on the drugs that do not have any essential targets.

### Constructing a high-quality comprehensive protein-protein interactome network and a three-dimensional structurally resolved network

We compiled a list of human protein-protein interactions combining high-throughput high-quality yeast two-hybrid interaction datasets [28]–[31] with six major protein-protein interaction databases [32]–[37]. Since literature-curated interactions could contain low-quality interactions [38], [39], we filtered the dataset by applying the criteria that each interaction has to be either from a high-throughput high-quality experiment or supported by at least two independent publications. The interactome network contains 30,713 binary and co-complex interactions between 8,357 proteins. To evaluate network properties of drug targets, we mapped them to the high-quality protein-protein interactome network and calculated their network properties.

To reconstruct the three-dimensional (3D) structurally resolved network, we further filtered the interactions with binary evidence codes, since the concept of interaction interface does not apply when two proteins do not bind each other directly [12]. We then constructed the 3D-interaction network based on known co-crystal structures in the Protein Data Bank (PDB) [40] using a homology modeling approach as described earlier [12]. This approach has been demonstrated to be very effective and accurate in inferring protein-protein interaction interfaces [12]. The resulting structurally resolved protein interactome is composed of 6,594 interactions between 3,630 proteins.

### Curating a list of known disease associated genes

We compiled a list of diseases for each drug based on the “indication” field from the DrugBank database. For each drug, we then obtained the disease-associated genes for these diseases from the disease-gene association map we compiled earlier based on OMIM and HGMD databases [12], [41], [42]. We then calculated the average shortest distance on the binary interactome network for 1) pairs of target proteins and the genes associated with the “indicated” diseases and 2) pairs of target proteins and all other disease-associated genes (Figure 4).

### Calculation of shared interaction interfaces

For each drug target protein *T* that can be mapped to the structurally resolved network with at least two interaction partners, we measured the proportion of shared interaction interfaces by calculating the Jaccard similarity coefficient [43]:
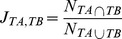
where 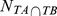 is the number of interacting domains on drug target protein *T* involved in both *T-A* and *T-B* interactions, and 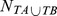 is the number of interacting domains involved in either *T-A* or *T-B* interaction. The mean of the Jaccard similarity coefficient was taken when a target protein has more than two interaction partners. To minimize potential confounding effects of essentiality, we analyzed the drugs with only one non-essential target to evaluate the effects of shared interaction interfaces of a drug target on the number of side effects.

### Bootstrapping approach for comparison of median number of side effects between different drug categories

While the vast majority of drugs have average distances between their targets and corresponding disease genes comparable to network mean distance (mean distance = 4.4), there are some drugs enriched with much smaller distances (distance<3; Figure 4A). We categorized the drugs into two classes using an average distance of 3 as cutoff to compare the median number of side effects. We carried out the bootstrapping approach to evaluate the difference of median number of side effects due to the observation of extremely unequal sample sizes (12 drugs with distance less than 3 and 319 drugs with distance equal to or bigger than 3) and variances between the two classes. For each drug class, we randomly sampled 10 observations with replacement and generated the median of these observations. The procedure was repeated 1000 times to obtain distributions of median number of side effects for each of the two drug classes. Then the Wilcoxon rank-sum test was used to evaluate the differences of median drug side effects between the two drug classes (Figure 4B). By randomizing the protein-protein interactions, the disease gene sets, and the drug target sets, we demonstrated that the observation is not due to potential biases in the data (Figure S7).

## Supporting Information

Figure S1 Generalized linear regression for the number of drug side effects over the FDA approval dates of drugs suggests an increasing trend in the number of side effects.
(EPS)

Figure S2 (**A**) The number of drug side effects is positively correlated with the number of essential targets. (**B**) The number of side effects is not correlated with the number of total targets for drugs with no essential targets.
(EPS)

Figure S3 The number of drug side effects is positively correlated with the number of bottlenecks with the cutoff of betweenness at (**A**) top 5%, (**B**) top 10%, and (**C**) top 40%.
(EPS)

Figure S4 Analyses of drug side effects for cancer drugs in terms of (**A**) the total number of targets, (**B**) the number of essential targets, (**C**) the number of targets for drugs with no essential targets, (**D**) average target degree, and (**E**) the number of bottleneck targets with betweenness at top 10%. Poisson model was used to address the effect of average target degree. Here the degree analysis is not limited to the drugs with only one non-essential target due to lack of data points.
(EPS)

Figure S5 Analyses of drug side effects for non-cancer drugs in terms of (**A**) the total number of targets, (**B**) the number of essential targets, (**C**) the number of targets for drugs with no essential targets, (**D**) target degree, and (**E**) the number of bottleneck targets with betweenness at top 10%. Poisson model was used to address the effect of the target degree.
(EPS)

Figure S6 Distribution of the proportion of shared interaction interface for (**A**) non-hub targets (degree<5) and hub targets (degree≥5), and (**B**) non-bottleneck targets and bottleneck targets (betweenness at top 20%).
(EPS)

Figure S7 Median number of side effects for the two drug classes from 100 randomization tests: (**A**) randomize protein-protein interactions; (**B**) randomize drug target sets; (**C**) randomize disease-gene associations. Error bars are standard errors. For network randomization, the edges of any two randomly selected interactions were swapped. Drug-target and disease-gene associations were randomly swapped.
(EPS)
